# Recent Advances in *Citrus maxima* and Its By-Products: Chemical Composition, Bioactivities, Safety, and Industrial Applications

**DOI:** 10.3390/foods15203646

**Published:** 2026-10-14

**Authors:** Le Li, Zhiqing Liu, Siting Wang, Quanhong Li

**Affiliations:** 1 School of Environmental and Quality Testing, Chongqing Chemical Industry Vocational College, No. 2009, East Bodhi Road, Changshou District, Chongqing 401228, China; 2 College of Food Science & Nutritional Engineering, China Agricultural University, No. 17 Tsinghua Dong Road, Beijing 100083, China; 18278289320@163.com; 3 School of Food Science and Engineering, South China University of Technology, No. 381 Wushan Road, Guangzhou 510640, China; 18278289375@163.com

**Keywords:** bioactive compounds, *Citrus maxima*, health benefits, industrial applications, nutritional components

## Abstract

*Citrus maxima*, the largest-fruited *Citrus* species, yields pulp and industrial by-products rich in bioactive phytochemicals with notable food and pharmaceutical potential. Yet its literature remains fragmented, lacking an integrated view of metabolite profiles, pharmacological spectrum, safety liabilities, and translational roadmaps. This Review consolidates nutritional composition, secondary metabolite diversity, and tissue-specific distribution across fruit and residues, critically appraises processing technologies, and maps applications spanning functional foods, edible packaging, nutraceuticals, pharmaceuticals, and agricultural inputs. Over 100 compounds, flavonoids, phenolic acids, coumarins, limonoid triterpenoids, acridone alkaloids, and volatile terpenes, underpin antioxidant, anti-inflammatory, antimicrobial, antitumor, antidiabetic, anti-obesity, hepatoprotective, gastroprotective, and neuroprotective effects. Toxicological data suggest pulp safety; however, furanocoumarin-mediated CYP3A4 inhibition is cultivar-dependent, as exemplified by higher furanocoumarin loads in pink/red-fleshed cultivars and ‘Guanximiyou’ than in white-fleshed types, warranting careful evaluation. Industrially, residues have been piloted in dietary fibre fortifiers, pectin extraction, craft brewing, edible films, bio-organic fertilizers, and botanical pesticides. By synthesizing dispersed findings and pinpointing gaps, cultivar-specific metabolomic variation, standardized extraction, and long-term safety, this Review bridges fundamental phytochemistry with bioprocess engineering and regulatory science, guiding high-value, sustainable utilization of this underutilized citrus resource.

## 1. Introduction

*Citrus maxima* (*C. maxima*) is the largest-fruited species in the genus *Citrus* (family *Rutaceae*) and is native to Southeast Asia and southern China, with a cultivation history spanning over a millennium [1,2]. The fruit is highly favored by consumers for its distinctive flavour and rich nutritional profile, and it is now widely cultivated in tropical and subtropical regions across the globe, demonstrating broad adaptability to diverse climatic and soil conditions. In China, the major *C. maxima*-producing provinces, Fujian, Guangdong, Guangxi, Sichuan and Zhejiang, benefit from warm, humid climates and abundant sunshine, which provide favourable ecological conditions for high-quality and high-yield production. The harvest season generally extends from September to December, with cultivar-specific variations in maturation time [2]. To date, numerous cultivars have been identified, among which the commonly grown ones include ‘Shatian’, ‘Wendan’, ‘Guanxi Honey’, ‘Changshou’, ‘Jinyou’ and ‘Hongrou’ (red-fleshed) *C. maxima*, exhibiting marked inter-cultivar diversity in fruit size, colour, flavour and nutrient composition [2,3]. In recent years, research on *C. maxima* has grown steadily, with particular emphasis on its bioactive constituents, such as flavonoid polyphenols, limonoids and essential oils, and their functional properties, including antioxidant, anti-inflammatory, antitumor and hypoglycaemic activities. This has positioned *C. maxima* as a research hotspot in food science and nutrition [4,5].

The *C. maxima* fruit is rich in essential nutrients, including proteins, carbohydrates, dietary fibre, organic acids, vitamins, and minerals [6,7]. Notably, it also contains a diverse array of bioactive compounds, such as flavonoids, coumarins, limonoid triterpenoids, acridone alkaloids, and volatile terpene essential oils [5,8]. These phytochemicals confer a broad spectrum of pharmacological effects, encompassing antioxidant, anti-inflammatory, antimicrobial, antitumor, antidiabetic, anti-obesity, hepatoprotective, and gastroprotective activities [5,9]. Some constituents have also indicated neuroprotective and cardiovascular protective properties [1,10]. Furthermore, the peel essential oil imparts a characteristic aroma to the fruit and is predominantly composed of terpenes, with limonene often accounting for more than 90% of the total relative abundance, along with β-pinene and γ-terpinene [11,12]. In addition to the bioactive compounds present in the pulp, the processing by-products, namely the flavedo, albedo, segment membranes and seeds, are likewise rich in dietary fibre, pectin and flavonoids, and exhibit multiple bioactivities such as antioxidant, antimicrobial and hepatoprotective effects. This makes *C. maxima* an economically valuable plant with high potential for whole-fruit utilization [13,14,15]. In traditional medicine, *C. maxima* peel and seeds have been used to improve digestion, relieve cough, and alleviate inflammation [5,16]. In recent years, the application of *C. maxima* by-products in functional food development has also attracted increasing attention, for example, using peel dietary fibre as a functional ingredient and naringin-rich extracts as raw materials for nutraceuticals [1,2]. This trend is consistent with the global consumer shift towards functional foods and natural health products. Beyond its nutritional and health-promoting value, *C. maxima* is a multipurpose economic crop with potential applications in food processing, pharmaceuticals and nutraceuticals, edible packaging materials, and agricultural inputs [5,13,14]. In the food industry, *C. maxima* holds considerable promise as a natural and healthy raw material, suitable for processing into juices, jams, preserves, dietary supplements, and functional beverages [2,17].

Despite these advances, recent studies on natural compounds in *C. maxima* and its by-products remain fragmented across disciplines, and several critical issues have not been systematically resolved. First, the chemical diversity of *C. maxima* is still incompletely mapped, particularly with respect to minor flavonoids, coumarins, limonoids, alkaloids, and volatile constituents that may differ substantially among cultivars, tissues, and geographical origins. Second, the tissue-specific accumulation and spatial distribution of bioactive compounds in the pulp, flavedo, albedo, segment membranes, and seeds are not fully understood, limiting precision-oriented utilization of different fruit parts. Third, although multiple bioactivities have been reported, the underlying molecular mechanisms, structure–activity relationships, synergistic interactions among phytochemicals, and dose–response relationships require further clarification. Fourth, safety evaluation, bioavailability, metabolic fate, and potential interactions with drugs or food matrices remain insufficiently investigated, which hinders clinical translation and functional food development. Fifth, industrial-scale extraction, purification, stabilization, and formulation technologies for *C. maxima*-derived bioactive compounds are still limited, and scalable, green, and cost-effective strategies are urgently needed. These gaps are particularly important because *C. maxima* by-products, including peel, segment membranes, and seeds, are generated in large quantities during juice processing and represent an underutilized reservoir of high-value natural compounds.

This Review therefore aims to analyse the botanical characteristics, chemical composition, nutritional value, health benefits, and underlying molecular mechanisms of *C. maxima* fruit and its derivatives, while also providing a thorough evaluation of their current applications in the food industry and other sectors. By comprehensively evaluating the chemical diversity, tissue-specific accumulation patterns, pharmacological activities, and processing technologies, this Review seeks to fill the aforementioned gaps. It not only offers references for cultivar selection and high-value utilization of by-products, but also explores the potential for developing functional foods, natural medicines, and green packaging materials, thereby contributing to the sustainable development and circular economy transformation of the *C. maxima* industry.

## 2. Nutritional Composition of *C. maxima* and Its Secondary Metabolites

The edible pulp of *C. maxima* contains approximately 89% water and provides only 38–42 kcal per 100 g of edible portion, characterizing it as a low-calorie, low-fat food [13,14]. Recent advances in food nutrition and metabolomics have enabled increasingly comprehensive profiling of its nutritional components, drawing substantial interest in both dietary nutrition and functional food development.

### 2.1. Proteins and Amino Acids

The protein content of *C. maxima* pulp is relatively low, ranging from 0.5 to 0.8 g/100 g edible portion. Despite this modest level, the amino acid profile is remarkably complete, with 18 detectable amino acids covering all essential ones required by humans, including valine, leucine, isoleucine, threonine and phenylalanine [18]. Notably, the distribution patterns of total hydrolysed amino acids and free amino acids differ considerably. Among free amino acids, proline is the most abundant, followed by aspartic acid and arginine. Of particular interest is the presence of physiologically active free amino acids such as gamma-aminobutyric acid (GABA), which provides a chemical basis for potential functional bioactivities. Furthermore, total amino acid content varies significantly across cultivars: for example, some honey *C. maxima* varieties contain approximately 4.53 g/100 g, whereas certain high-accumulating genotypes reach up to 7.04 g/100 g [18], indicating that genetic background exerts a profound regulatory influence on protein metabolism and accumulation. Extending the analysis to peel tissues, the crude protein content is substantially higher than that of the pulp, with values of 8.23% and 7.67% recorded for white-fleshed and red-fleshed *C. maxima* peels, respectively. This highlights the untapped potential of processing by-products as alternative protein sources.

### 2.2. Carbohydrates

Carbohydrates constitute the major macronutrient fraction in *C. maxima* pulp, comprising both soluble sugars and dietary fibre (including pectin). With respect to soluble sugar composition, sucrose, fructose and glucose are the predominant saccharides in *C. maxima* juice. Based on a mean analysis of 24 cultivars, Sharma et al. [2] reported that these three sugars account for approximately 48%, 26% and 25% of total soluble sugars, respectively, with glucose and fructose being directly absorbable by the human body. Notably, sugar accumulation profiles exhibit marked cultivar-dependent patterns; for instance, the cultivar ‘Hongroumi 1’ displays the highest total sugar and sucrose contents (55.28 mg/g) among the tested varieties. In contrast to soluble sugars, dietary fibre in the pulp is relatively low, at only about 1 g/100 g edible portion, with insoluble fibre predominating and accounting for 60–80% of the total fibre [13,14]. In striking contrast, dietary fibre levels in the peel are far higher, with reported values ranging from 65.72% to 89.64%, of which soluble and insoluble fractions constitute approximately 15–36% and 43–53%, respectively [6,19]. As a major soluble polysaccharide component, pectin is structurally composed of a galacturonic acid backbone [6]. The dominance of insoluble fibre, especially in the pulp, effectively retards the intestinal absorption rate of sugars, thereby contributing to the fruit’s low glycaemic index, which provides a structural basis for its low glycaemic response characteristics [2].

### 2.3. Vitamins and Minerals

Vitamin C is one of the representative micronutrients in *C. maxima* pulp, with contents varying considerably depending on cultivar and geographical origin, commonly reported in the range of 21.0–78.4 mg/100 g [2]. A determination across 24 cultivars by Sharma et al. [2] further suggested that this variability is cultivar-dependent, with premium varieties such as ‘Khao Yai’ exhibiting up to 78.4 mg/100 g in freshly squeezed juice, and ‘Tahitian’ also ranking among the high-content types, underscoring the primary role of genetic background in driving this trait. Beyond vitamin C, *C. maxima* pulp also contains a spectrum of other vitamins, including B_1_ (thiamine), B_2_ (riboflavin), B_12_, vitamin A and vitamin E [2,20], albeit at lower levels than vitamin C, yet collectively conferring a relatively comprehensive profile of both water-soluble and fat-soluble vitamins. Notably, vitamin C, as a potent antioxidant, acts synergistically with the aforementioned dietary fibre (which delays sugar absorption) and GABA (a bioactive amino acid) to collectively underpin the health value of *C. maxima*, characterized by low glycaemic response, antioxidant capacity and multi-component synergy.

The mineral composition of *C. maxima* is notably distinguished by a high-potassium and low-sodium profile, with potassium content ranging from 150 to 250 mg per 100 g pulp and sodium only 1–3 mg/100 g, a ratio that confers potential value in dietary blood pressure management [19,21]. Among macroelements, calcium, magnesium and phosphorus fluctuate in the ranges of 10–30 mg/100 g, 10–15 mg/100 g and 15–20 mg/100 g, respectively [21,22]. For trace elements, iron (0.10–0.50 mg/100 g), zinc (0.10–0.30 mg/100 g), selenium (1–5 μg/100 g), copper (0.05–0.10 mg/100 g) and manganese (0.02–0.05 mg/100 g) have all been detected, with iron and copper levels being relatively high among citrus fruits [21]. Importantly, the above mineral contents are subject to dual synergistic regulation by cultivar genetic background and tissue-specific spatial distribution. At the cultivar level, inter-individual differences can substantially expand the value range; for example, calcium content in certain varieties has been reported to reach 60 mg/100 g, far exceeding the typical average [19]. At the tissue level, the peel exhibits a far greater capacity for mineral enrichment than the pulp, with sodium content approximately three times and potassium about 50% higher than those in the pulp [21]. Further sub-layer analysis reveals that these mineral elements are primarily chelated in the flavedo (outer coloured layer), whereas their concentrations in the albedo (inner white layer) are significantly lower, demonstrating a highly ordered tissue-specific gradient distribution pattern [21]. This progressive spatial delineation, from cultivar to tissue to sub-layer, provides a precise structural framework for nutritional evaluation of *C. maxima* minerals and for comprehensive utilization of its processing by-products.

## 3. Secondary Metabolites of *C. maxima* and Its By-Products

Beyond the macronutrients (carbohydrates and proteins) and micronutrients (vitamins and minerals) discussed above, the nutritional value of *C. maxima* is more extensively manifested in its remarkably diverse array of secondary metabolites. Unlike primary metabolites, these compounds are not directly essential for basic life-sustaining processes, yet they play critical adaptive roles in plant stress responses, pathogen defence and ecological interactions.

### 3.1. Polyphenols

**Figure 1:**
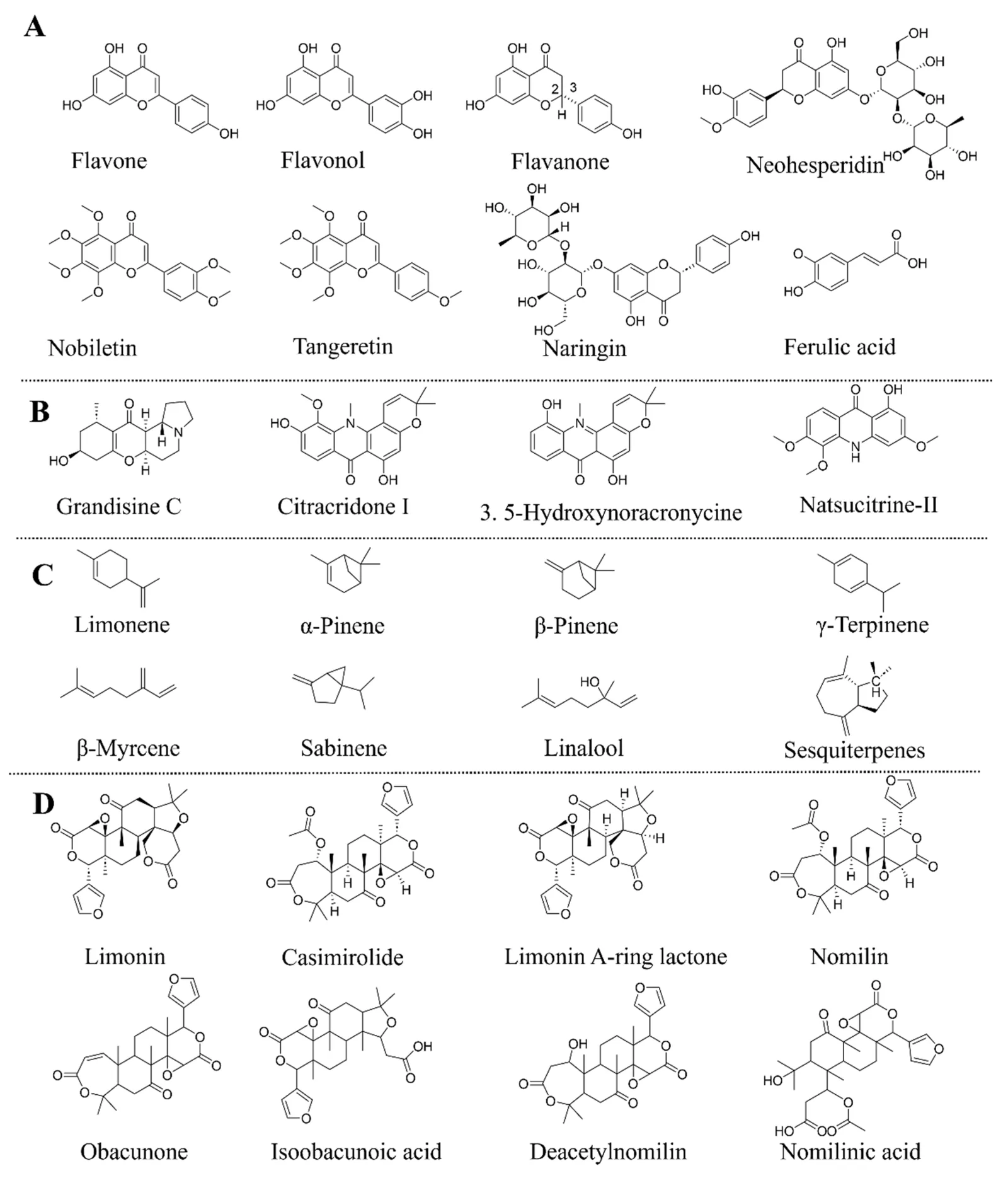
Secondary metabolites of *C. maxima* and its by-products. (**A**) Polyphenols (**B**), alkaloids (**C**), volatile compounds, and limonoids and other terpenoids (**D**).

Among the vast secondary metabolite repertoire of *C. maxima*, polyphenols represent the most abundant and most extensively investigated class. Based on chemical structure, they can be subdivided into two major subclasses, flavonoids and phenolic acids, with flavonoids being the predominant group (Figure 1A, Table 1). Flavonoids share a common C_6_–C_3_–C_6_ carbon skeleton and are further classified into flavones, flavonols, and flavanones according to the oxidation state of the C-ring and substitution patterns. In *C. maxima* peel, flavanones overwhelmingly dominate the flavonoid profile, with naringin (naringenin-7-O-neohesperidoside) alone accounting for more than 80% of the total flavanone content and reaching concentrations as high as 5–15% of the peel dry weight [2]. At the tissue level, naringin is not homogeneously distributed but is highly concentrated in the exocarp (50–70%), the whole peel (25–40%) and the segment membrane (5–15%), and its levels decline progressively with fruit ripening, suggesting a developmental stage-dependent regulatory mechanism [2]. In addition to naringin, other flavanone glycosides such as neohesperidin and narirutin have also been identified in *C. maxima* [8]. Notably, polymethoxylated flavones, including nobiletin and tangeretin, although present at much lower concentrations than naringin, have recently emerged as frontiers of functional research owing to their potent anti-inflammatory, anticancer, and neuroprotective activities [2,9].

**Table 1:** Bioactive compounds in *C. maxima* and its by-products.

Parts	Phytochemicals	Identification Techniques	Chemical Compositions	References
Fuits	Sugars, alcohols, phenolic acids, organic acids, amino acids, lipids, vitamins	UPLC-MS/MS	P-coumaric acid, caffeic aldehyde, caffeic acid, ferulic acid; downregulated, including cinnamic acid, vanillic acid, coniferaldehyde, succinic semialdehyde, phenylpyruvic acid, 2-propylmalic acid, L-ornithine, L-tyrosine, L-tryptophan); Sugars and alcohols, Lipids; Vitamins	[18]
Peel	Flavonoids (flavanones, polymethoxyflavones), essential oils (monoterpenes), carotenoids, limonoids, pectin	HPLC, GC-MS, UPLC-MS/MS	Hsperidin, narirutin, neohesperidin; Malic acid, tartaric acid, quinic acid; glucose, fructose; L-ascorbic acid; β-carotene, β-cryptoxanthin, zeaxanthin	[23]
Juice	Flavonoids (glycosides), vitamin C, organic acids, sugars, volatile aroma compounds	HPLC, GC-MS	Hesperidin, narirutin, didymin; Citric acid, malic acid; sucrose, glucose, fructose; L-ascorbic acid; limonene, linalool, α-pinene, myrcene	[23]
Seeds	Limonoids (aglycones), flavonoids, fatty oils	HPLC, GC-MS	Limonin, nomilin, obacunone; linoleic acid, oleic acid, palmitic acid, stearic acid	[23]
Peel	Phenolic acids, flavonoids, anthocyanins, tannins	HPLC,	Hsperidin, Narirutin, Nringin	[2]
Peel	Essential oils, dietary fibers, polyphenols, flavonoids	HPLC, GC-MS	Naringin, narirutine, D-limonene, γ-terpinene, β-pinene, α-pinene, β-sitosterolstigmasterol	[13]
Peel	Essential oils, Flavonoids	GC-MS	Limonene, α-pinene, camphene, myrcene, naringin	[20]
Pulp	Phenolic compounds, Flavonoids	HPLC	Maleic acid, ferulic acid, tannic acid; naringin, kaempferol, rutin, quercetin, saponins	[20]
Seeds	Phenolic compounds, Flavonoids	HPLC	Apigenin	[20]
Pulp	Minerals	ICP-OES	Na^+^, K^+^, Ca^2+^, Mg^2+^, Fe^2+^, Zn^2+^, Cu^2+^, Mn^2+^, Se^2+^	[21]
Peel	Minerals	ICP-OES	Na^+^, K^+^, Ca^2+^, Mg^2+^, Fe^2+^, Zn^2+^, Cu^2+^, Mn^2+^, Se^2+^	[21]
Fruit	Sugars, organic acids, vitamin C, mineral elements	-	Sucrose, Fructose, Glucose, Citric acid, Quinine; Vitamin C; Na^+^, K^+^	[24]

As another major component of polyphenols, phenolic acids are abundant in *C. maxima* juice, with ferulic acid as a representative monomer [25]. Studies using ‘Shatian’ *C. maxima* as a model have further revealed that both free and bound phenolics coexist in different tissues, including the oil gland layer, albedo, segment membrane and seeds, and that the phenolic composition and antioxidant activity vary significantly among these tissues [2]. Although the absolute content of phenolic acids is lower than that of flavonoids, their synergistic antioxidant effects with flavonoids have been experimentally suggested to be non-negligible [26]. Therefore, a comprehensive evaluation of the overall antioxidant capacity of *C. maxima* should take into account the combined contributions of both flavonoid and phenolic acid fractions.

### 3.2. Alkaloids

Among the secondary metabolite spectrum of *C. maxima*, alkaloids represent a group of nitrogen-containing compounds that are relatively low in abundance yet structurally distinctive, with acridone alkaloids being the most representative [5]. These natural products, characterized by an acridan-9-one core structure, hold significant chemotaxonomic value within the *Rutaceae* family and are often regarded as one of the chemical fingerprints distinguishing this family from other plant groups (Figure 1B, Table 1) [5]. To date, several acridone alkaloids, including grandisine and citracridone, have been identified from *C. maxima* fruits [5,27]. Despite their substantially lower absolute content compared to the major polyphenolic constituents, acridone alkaloids have attracted considerable interest owing to their diverse pharmacological activities, including antimicrobial, antitumor, and anti-inflammatory properties [5]. It is worth emphasizing that, relative to other *Citrus* species, investigations into the alkaloid profile of *C. maxima* remain comparatively limited [5]. Current knowledge is primarily derived from preliminary targeted metabolomic analyses, while fundamental questions, such as tissue-specific distribution patterns among peel, pulp, and seeds; inter-cultivar accumulation differences; developmental dynamics during maturation; and the identification of key biosynthetic enzymes, await more in-depth elucidation. With the maturation of high-resolution mass spectrometry and single-cell/spatial metabolomics technologies, the global characterization of low-abundance alkaloid components and the delineation of their regulatory networks in *C. maxima* are expected to become important breakthroughs in this field.

### 3.3. Volatile Compounds

Volatile compounds constitute the chemical basis of the characteristic aroma of *C. maxima* and are the core components of *C. maxima* essential oil. Chemically, terpenes predominate, with the monoterpene limonene alone accounting for more than 90% of the relative content in peel essential oil, along with other monoterpenes such as β-pinene and γ-terpinene, as well as various sesquiterpenes (Figure 1C, Table 1). In terms of tissue distribution, volatiles display a highly heterogeneous pattern: the peel, particularly the oil glands in the exocarp, serves as the primary site of biosynthesis and storage, with essential oil contents (approximately 0.5–1.5% on a dry weight basis) significantly higher than those in the pulp, which lacks oil gland structures and consequently contains extremely low volatile levels [28]. The chemical composition and content of peel essential oil are co-regulated by multiple hierarchical factors, including intrinsic cultivar genetic background, as well as extrinsic variables such as fruit maturity, growing environment and post-harvest treatment [4,11].

Functionally, the volatile terpenes of *C. maxima* play a dual role as both “quality determinants” and “ecological chemical defence agents”. On one hand, the complex aroma profiles they generate serve as important indicators of fruit sensory quality; on the other hand, upon pathogen attack or mechanical damage, the induced release of volatile compounds can act as defence signals involved in stress responses, exerting broad-spectrum bioactivities including antimicrobial, antiparasitic, anti-inflammatory and antioxidant effects [4,12]. In recent years, the iterative application of high-resolution analytical techniques such as gas chromatography-mass spectrometry (GC-MS) and comprehensive two-dimensional gas chromatography (GC × GC) has substantially enhanced the precision of qualitative and quantitative analyses of *C. maxima* volatiles, providing robust methodological support for the accurate characterization of aroma quality across cultivars, the screening of characteristic markers, and the early warning of post-harvest flavour deterioration [11,28].

### 3.4. Limonoids and Other Terpenoids

Limonoids represent a structurally highly diverse class of triterpenoid compounds in the secondary metabolite repertoire of *C. maxima*. Chemotaxonomically, limonoids are highly oxygenated tetranortriterpenoids predominantly distributed in the *Rutaceae* and *Meliaceae* families, and *C. maxima* is regarded as one of the important natural sources of these compounds (Figure 1D, Table 1). In contrast to the flavonoids/phenolic acids derived from the phenylpropanoid pathway and the nitrogen-containing acridone alkaloids discussed above, limonoids are biosynthesized via the mevalonate (MVA) pathway, representing an independent branch of triterpenoid secondary metabolism in the chemical diversity of *C. maxima*.

With respect to tissue distribution, a total of 21 limonoids have been identified across the five major anatomical structures of *C. maxima*, flavedo, albedo, segment membrane, juice vesicles and seeds, but with remarkable inter-tissue heterogeneity [15]. Seeds are the most enriched site for limonoid accumulation, harbouring up to 14 distinct limonoids; in contrast, the flavedo accumulates only three major limonoids, namely limonin, casimirolide and limonin A-ring lactone, indicating that seeds are not only storage organs but also active centres of limonoid metabolism [15]. Importantly, the distribution of limonoids across tissues is not an isolated phenomenon: significant positive correlations exist between juice vesicles and seeds, as well as between albedo and segment membrane, suggesting possible transport of limonoids or shared precursor metabolic flux among adjacent or functionally related tissues [15].

From a functional perspective, limonoids exhibit a “double-edged” effect, spanning both the pharmaceutical/health and food processing domains. In terms of pharmacological activities, representative limonoids such as limonin and nomilin have been reported to possess antitumor, antimicrobial, anti-inflammatory, analgesic, antiviral, insect antifeedant, and sedative properties [15]; more recent studies have further revealed their potential in antioxidant, hepatoprotective, and neuroprotective effects [1]. Concurrently, in the food science arena, limonoids are important chemical contributors to the “delayed bitterness” of *C. maxima* juice, a bitterness that does not appear immediately but gradually develops within hours after juicing, owing to the enzymatic ring-opening of limonin A-ring lactone. This characteristic poses a substantial challenge to the flavour quality and consumer acceptance of processed *C. maxima* products, making precise regulation of limonoid content a key target in processing optimization [1,15]. Overall, limonoids play an irreplaceable cross-cutting role in the chemical defence ecology of *C. maxima*, the development of functional foods, and the quality control of processing operations.

## 4. Health Benefits Conferred by Bioactive Constituents of *C. maxima* and Its By-Products

The fruit of *C. maxima* and its processing by-products have been established as natural co-enrichment systems for a diverse array of bioactive compounds, including flavonoids, polysaccharides, coumarins, limonoid triterpenoids, and volatile terpene essential oils. This multifaceted chemical repertoire not only serves as important chemotaxonomic markers but has also been progressively elucidated at the molecular pharmacological level as functional molecular ensembles with defined signal-pathway targeting. Modern pharmacological studies indicate that the aforementioned bioactive components exert dose-dependent interventions across multiple pathophysiological processes, encompassing free-radical scavenging, regulation of inflammatory cascades, inhibition of pathogenic microorganisms, suppression of tumour cell proliferation, modulation of glucolipid metabolic homeostasis, repair of hepatocyte injury and protection of the gastrointestinal mucosa.

### 4.1. Antioxidant Activity

**Figure 2:**
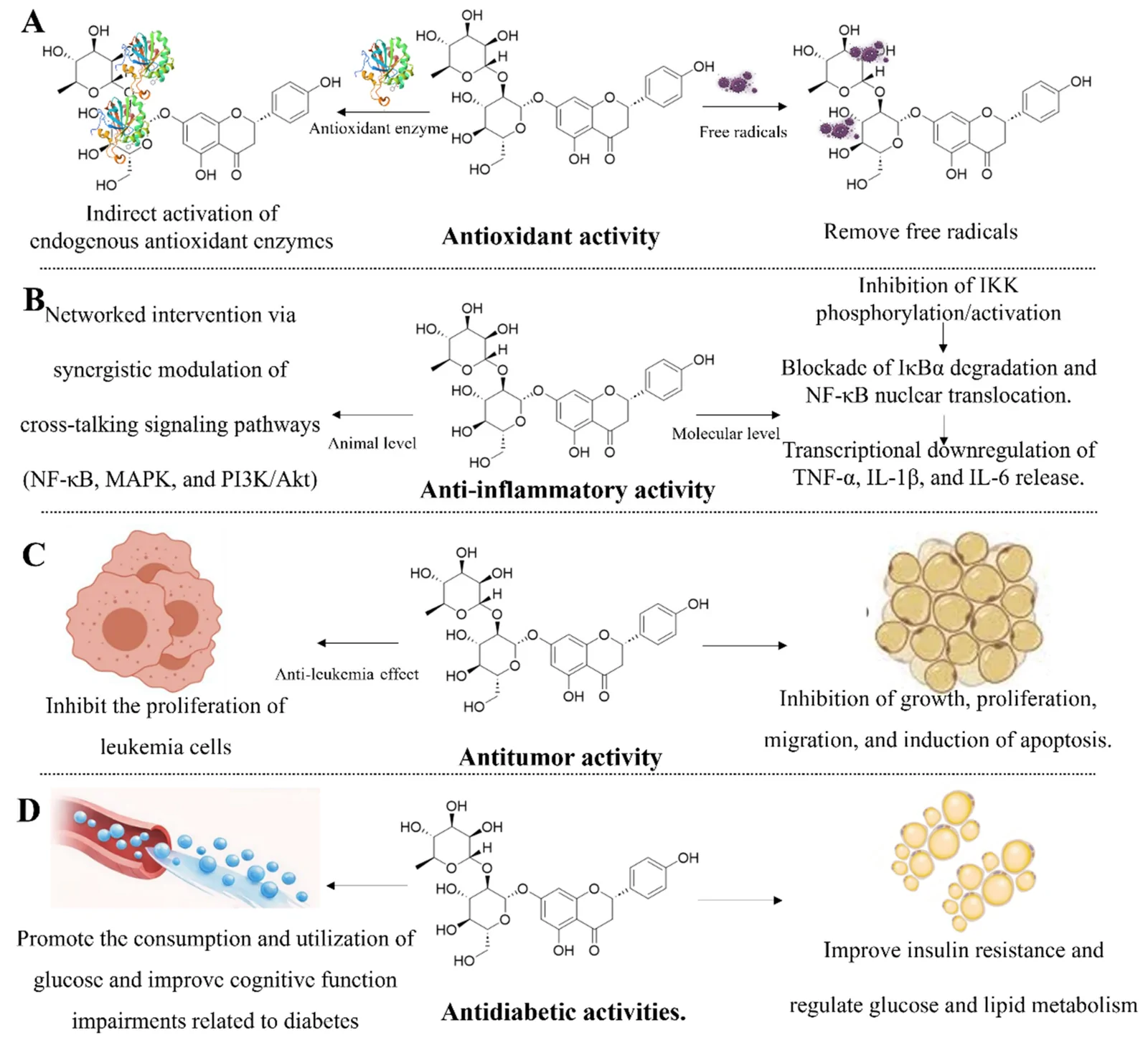
(**A**) Antioxidant, (**B**) anti-inflammatory, (**C**) antitumor, and (**D**) antidiabetic activities.

Antioxidant activity represents the most extensively investigated and mechanistically foundational aspect of the multiple health benefits of *C. maxima*, rooted in a synergistic chemical defence network composed of flavonoids (predominantly naringin and its aglycone naringenin), phenolic acids, vitamin C and volatile terpenes (Table 2) [6,25]. At the molecular level, this antioxidant system operates through at least two parallel pathways. The first is direct free-radical scavenging based on structural chemistry. Flavanones such as naringin, owing to the presence of ortho-dihydroxyl groups on the B-ring and the C2=C3 double bond conjugated with the 4-oxo moiety, are endowed with the chemical capacity to donate hydrogen atoms or electrons, thereby effectively quenching stable radicals such as DPPH and ABTS, and reducing ferric ions (FRAP). Their scavenging activities against hydroxyl radicals (·OH) and superoxide anions (O_2_·^−^) exhibit clear concentration-dependent relationships (Figure 2A) [6,29]. The second is indirect activation of endogenous antioxidant enzyme systems via signal transduction. Naringin, for instance, can interact with redox-sensitive thiol groups of the Keap1 protein, promoting the dissociation and nuclear translocation of transcription factor Nrf2 from the Keap1–Nrf2 complex. Upon binding to antioxidant response elements (AREs), this pathway drives the transcriptional upregulation of downstream phase II detoxifying and antioxidant enzymes, including superoxide dismutase (SOD), catalase (CAT) and glutathione peroxidase (GPx) [10]. This route not only eliminates already-generated reactive oxygen species but also enhances the intrinsic antioxidant reserve capacity of cells, constituting a positive feedback loop of “warning–response–amplification”.

**Table 2:** Health-promoting effects of *C. maxima* and its by-products.

Parts	Assays	Biological Activity	Main Findings	References
Peel	In vitro	Antioxidant activity	1. The oils displayed concentration-dependent DPPH radical-scavenging activity.	[29]
Peel	In vitro	Antioxidant activity	1. The ethanolic peel extract had relatively higher total phenolic and total flavonoid contents than the other extracts. 2. The ethanolic peel extract showed the most prominent antioxidant activity, suggesting that *C. maxima* peel polyphenols are a good source of natural antioxidants.	[25]
Fruits	In vitro	Antioxidant activity	1. Preliminary phytochemical analysis revealed the presence of triterpenoids, steroids, glycosides, alkaloids, and flavonoids. 2. Exhibited concentration-dependent radical-scavenging activity and reducing power.	[25]
Fruits	In vitro	Antioxidant activity	1. Total phenolics, tannins, and total flavonoids ranged from 836.90–909.52 mg GAE/L, 333.33–523.21 mg GAE/L, and 224.88–262.22 mg rutin equivalent/L, respectively. 2. All juice samples exhibited strong antioxidant activity in multiple in vitro assays. 3. The juices indicated potent anti-diabetic potential through significant inhibition of α-amylase (75.55–79.75%) and α-glucosidase (70.68–72.83%). 4. All samples showed excellent inhibitory activity against tyrosinase (76.95–80.79%), acetylcholinesterase (75.71–79.74%), and β-glucuronidase (68.13–69.38%). 5. The study concludes that fresh juice from *C. hystrix* and *C. maxima* (Red & White varieties) has potential as a source of antioxidant agents, functional foods, and nutraceuticals	[6]
Seed	In vitro	Antioxidant activity	1. Rich in antioxidant phytoconstituents: Total phenolic content (TPC) and HPLC analysis suggested that EESCM contains potent antioxidant compounds, including naringin, caffeic acid, and hesperidin. 2. Link between antioxidants and safety: The study suggests that the reasonably non-toxic profile of EESCM observed in animal experiments (e.g., no signs of toxicity in mice after a single oral dose up to 3000 mg/kg in the acute toxicity test) may be attributable to the presence of these strong antioxidant components.	[30]
Peel	In vitro	Anti-inflammatory	1. Ethnomedicinal basis: In traditional medicine, *C. maxima* has been used to treat ulcers, swelling, pain, and other inflammation-related conditions. 2. Bioactive constituents: The plant is rich in compounds with anti-inflammatory potential, including polyphenols, flavonoids, coumarins, and terpenoids. 3. Broad pharmacological actions: In addition to anti-inflammatory effects, it exhibits antioxidant, antimicrobial, analgesic, and anticancer activities, which may synergise with its anti-inflammatory action. 4. Research outlook: The article emphasises the need for further studies to explore the detailed molecular mechanisms underlying the anti-inflammatory effects of *C. maxima* extracts and their active compounds.	[5]
Peel	In vivo (125–500 mg/kg/day)	Anti-inflammatory	1. Animal model anti-inflammation: Methanolic seed extract of *C. maxima* (MECM) showed significant anti-inflammatory effects in formalin- and carrageenan-induced mouse paw oedema models. 2. Disease model intervention: Aqueous pulp extract improved inflammatory processes in non-alcoholic fatty liver disease (NAFLD) rats; ethanolic peel extract showed therapeutic potential in carrageenan-induced rat inflammation models. 3. Anti-inflammatory mechanism: The aqueous peel extract of Kawachi-bankan also inhibited the production of inflammatory cytokines such as IL-6 and TNF-α in vivo. 4. Formulation validation: The oil-loaded microspheres also indicated enhanced anti-inflammatory activity in in vivo models.	[5]
Seed	In vivo (200, 400 mg/kg/day)	Anti-inflammatory	1. Significant anti-inflammatory effect: MECM significantly inhibited mouse paw oedema in both formalin- and carrageenan-induced inflammatory models. 2. Clear dose–response relationship: MECM exhibited anti-inflammatory activity at doses of 200 mg/kg and 400 mg/kg. 3. Effect comparable to standard drug: Its anti-inflammatory effect was similar to that of the positive control drug indomethacin (10 mg/kg). 4. Well-validated models: Two classical inflammation models (formalin- and carrageenan-induced) were used to cross-validate the anti-inflammatory effect, strengthening the reliability of the conclusions. Defined phase of action: In the formalin-induced paw oedema test, MECM inhibited oedema in both the early and late phases.	[16]
Peel	In vitro	Antimicrobial	1. Negative antimicrobial result: No positive antimicrobial activity was observed in the antimicrobial assays conducted in this study. 2. Possible influencing factors: The authors attributed the negative antimicrobial results to several possible reasons, including resistance of the test strains, differences in preparation temperature conditions, and the relatively low concentration of phytochemicals in the plant extracts. 3. Extraction methods: The peel extracts of *C. maxima* were prepared using n-hexane as the solvent, employing both maceration and Soxhlet extraction methods. 4. Phytochemical screening: Qualitative detection suggested the presence of alkaloids in all extracts. Terpenoids were detected in the filtrate and residue from maceration, but not in the Soxhlet extracts. Flavonoids were found only in the residue.	[31]
Peel	In vitro	Antimicrobial	1. Broad-spectrum antibacterial activity: CmAgNPs indicated good antibacterial activity against *Staphylococcus aureus*, *Pseudomonas aeruginosa*, methicillin-resistant *Staphylococcus aureus* (MRSA), and *Escherichia coli*. 2. Prominent anti-MRSA activity: In the antimicrobial test against MRSA, the zone of inhibition reached 18.5 mm. 3. Antifungal activity: CmAgNPs exhibited zones of inhibition of 22.67 mm against Candida glabrata and 11 mm against *Candida albicans*. 4. Good biocompatibility: MTT assay revealed that CmAgNPs maintained cell viability above 75% in 3T3-L1 cells, indicating favourable biocompatibility. 5. Multifunctional bioactivity: In addition to antimicrobial effects, CmAgNPs also showed antioxidant activity (IC_50_ = 26.4 µg/mL in the DPPH assay), antidiabetic activity (improving glucose uptake by 49.15%), and anti-adipogenic activity (preventing lipid accumulation by 39%).	[32]
Peel	In vivo (25–200 mg/kg/day)	Antineoplastic	1. Clear antitumor effect: PTFC and its components can inhibit tumor progression. 2. Multi-target mechanism of action: PTFC exerts its antitumor effects through multiple pathways, including regulating glycolipid metabolism, and blocking peroxidation and persistent inflammation. 3. Broad therapeutic potential: The review indicates that PTFC shows promise in the prevention or treatment of hepatic metabolic diseases, cardiovascular diseases, intestinal barrier dysfunction, and malignancies.	[9]
Peel	In vitro	Antineoplastic	1. Clear cytotoxic effect: *C. maxima* extract increased the death of MDA-MB-231 breast cancer cells, with an IC_50_ value of 338 μg/mL at 24 h. 2. Mechanism of action—ROS induction: *C. maxima* extract inhibited cancer cell proliferation by inducing elevated levels of reactive oxygen species (ROS). Excessive ROS levels can activate apoptosis and inhibit cancer cell metastasis through the NF-κB pathway. 3. Identification of key active compound: Molecular docking studies revealed that the flavonoid compound hesperidin from *C. maxima* exhibited the best binding energy (−21.4766 kcal/mol) with the NF-κB protein. 4. Molecular-level interactions: The binding of hesperidin to the active site of the NF-κB protein suggested that amino acid residues (His537, Asp519, Gly407, Gln479, Arg416) may play a role in ROS metabolism.	[33]
Juice	In vitro	Antidiabetic	1. Significant α-amylase inhibition: The fresh juice of *C. maxima* showed α-amylase inhibition ranging from 75.55% to 79.75%. 2. Significant α-glucosidase inhibition: The juice exhibited α-glucosidase inhibition ranging from 70.68% to 72.83%. 3. Multi-target activity: In addition to anti-diabetic activity, the juice also indicated significant antioxidant activity (evaluated through various in vitro assays including DPPH•, ABTS•^+^, and •OH radical scavenging), tyrosinase inhibitory activity (76.95–80.79%), and acetylcholinesterase inhibitory activity (75.71–79.74%).	[6]
Peel	In vivo (200–600 mg/kg/day)	Antidiabetic	1. Significant hypoglycemic effect: The peel extract significantly reduced blood glucose levels in diabetic rats by 70.17% (*p* < 0.05). 2. Clear dose–response relationship: Three dose groups (200, 400, and 600 mg/kg body weight/day) were established, with continuous oral administration by gavage for 14 days. 3. Improved lipid profile: At the 600 mg/kg dose, the extract increased high-density lipoprotein cholesterol (HDL-C) by 4.43%, while simultaneously reducing total cholesterol (TC) by 30.86%, triglycerides (TG) by 10.58%, and low-density lipoprotein cholesterol (LDL-C) by 10.20%. 4. Hepatoprotective effect: Liver enzyme activities in the treatment groups showed no significant changes, whereas they were significantly elevated in the control group, suggesting that the peel extract possesses hepatoprotective potential.	[34]
Peel	In vitro	Antidiabetic	1. Improved glucose uptake: CmAgNPs improved glucose uptake by 49.15% in 3T3-L1 cells. Prevention of lipid accumulation: CmAgNPs prevented lipid accumulation by 39% in 3T3-L1 cells. 2. Gene expression regulation: RT-qPCR suggested that CmAgNPs upregulated the gene expression of GLUT4, PPAR-α, and adiponectin, while downregulating the expression of PPAR-γ and leptin. 3. Multi-target bioactivity: In addition to antidiabetic activity, CmAgNPs also exhibited potent antioxidant activity (IC_50_ = 26.4 µg/mL in the DPPH assay), antimicrobial activity (zone of inhibition of 18.5 mm against MRSA), and anti-adipogenic activity. 4. Good biocompatibility: MTT assay revealed that CmAgNPs maintained cell viability above 75% in 3T3-L1 cells, indicating favourable biocompatibility.	[32]
Leaves	In vivo (125–500 mg/kg/day)	Anti-obesity	1. In both olanzapine-induced and cafeteria diet-induced obese rat models, the ethanolic leaf extract exhibited significant anti-obesity activity. 2. Compared with the obese control group, the body weight, body temperature, and multiple serum parameters of the treated rats were significantly decreased (*p* < 0.05).	[5]
Peel	In vivo (125–500 mg/kg/day)	Anti-obesity	1. After intervention with the ethanolic peel extract, the body weight of the mice was significantly reduced. 2. The fasting blood glucose levels of the mice were markedly decreased. 3. The hepatic lipid content and serum insulin levels of the mice were also significantly lowered.	[5]
Leaves	In vivo (125–500 mg/kg/day)	Liver protection	1. Leaf extract ameliorates non-alcoholic fatty liver disease: The leaf extract of *C. maxima* exhibited hepatoprotective, hypolipidemic, anti-inflammatory, and antioxidant activities in a rat model of non-alcoholic fatty liver disease (NAFLD). 2. Pure total flavonoids (PTFC) prevent and treat hepatic metabolic diseases: The pure total flavonoids extracted from *C. maxima*, including naringin, hesperidin, narirutin, and neohesperidin, possess biological functions in the prevention or treatment of hepatic metabolic diseases. 3. Bioactive constituent basis: Flavonoids abundant in *C. maxima*, particularly naringin, are considered important material bases for its hepatoprotective activity.	[5]
Peel	In vivo (125–500 mg/kg/day)	Liver protection	1. Peel powder counteracts chemical-induced liver injury: In a carbon tetrachloride (CCl_4_)-induced liver injury rat model, dietary supplementation with *C. maxima* peel powder significantly reduced serum AST, ALT, and ALP activities, and markedly decreased oxidative stress markers (MDA, NO, and APOP levels), demonstrating significant hepatoprotective effects. 2. Pure total flavonoids (PTFC) prevent and treat hepatic metabolic diseases: The pure total flavonoids extracted from *C. maxima*, including naringin, hesperidin, narirutin, and neohesperidin, possess biological functions in the prevention or treatment of hepatic metabolic diseases. 3. Bioactive constituent basis: Flavonoids abundant in *C. maxima,* particularly naringin, are considered important material bases for its hepatoprotective activity.	[5]
Peel	In vivo (100–400 mg/kg/day)	Liver protection	1. Phytochemical analysis: The ethanolic peel extract of *C. maxima* contained secondary metabolites including flavonoids, tannins, alkaloids, triterpenoids, and quinones. 2. Significant reduction in liver enzymes: Compared with the negative control group (which received only isoniazid and rifampicin), all treatment groups receiving the peel extract showed significantly lower serum SGPT (ALT) and SGOT (AST) levels. 3. Clear dose–response relationship: Three dose groups were established at 100, 200, and 400 mg/kg body weight. The 400 mg/kg dose exhibited the best mitigating effect on elevated liver enzymes, demonstrating a dose-dependent protective effect.	[35]
Peel	In vivo (25–200 mg/kg/day)	Gastrointestinal Protection and Intestinal Microbiome Regulation	1. Protection of intestinal barrier integrity: PTFC and its components can protect the integrity of the intestinal barrier, which is of great significance for maintaining intestinal health and preventing harmful substances from entering the bloodstream. 2. Positive regulation of intestinal microbiota: PTFC and its components can positively regulate the intestinal microbiota and promote the balance of intestinal microecology. 3. Prevention and treatment of intestinal barrier dysfunction: The review summarised the biological functions of PTFC and its components in preventing or treating intestinal barrier dysfunction. 4. Multi-target synergistic effects: PTFC exerts its protective effects on the intestine through multiple pathways, including regulating glycolipid metabolism, and blocking peroxidation and persistent inflammation. 5. Influence of individual differences: The article pointed out that differences in fruit cultivation systems, harvesting standards, preparation methods, delivery systems, and individual intestinal microecology can all affect the specific therapeutic efficacy of PTFC.	[9]
Peel	In vivo (150 mg/kg/day)	Gastrointestinal Protection and Intestinal Microbiome Regulation	1. Improved defecation parameters: Supplementation with all three components (CEL, PEC, and CPP) improved defecation parameters in constipated mice. 2. Pectin regulates signaling pathways: PEC effectively regulated the SCF/C-kit and Nrf2/HO-1 signaling pathways. 3. Pectin modulates gut microbiota: Compared with the model group, PEC increased the abundance of Akkermansia by approximately 34% and decreased the abundance of Desulfovibrio by approximately 26%. 4. Physicochemical advantages of pectin: PEC exhibited smaller particle size and better solubility, which promoted the production of beneficial metabolites and thereby alleviated constipation. 5. Mechanism of cellulose action: CEL alleviated constipation primarily through its physical properties, such as increasing faecal bulk.	[36]
Peel	In vitro	Gastrointestinal Protection and Intestinal Microbiome Regulation	1. Promotion of Bifidobacterium growth: After co-fermentation with faecal samples, CPFEs from chachiensis and grapefruit significantly increased the abundance of Bifidobacterium spp., which was associated with the enrichment of naringin and hesperidin. 2. Increased short-chain fatty acid (SCFA) production: CPFEs from chachiensis and grapefruit stimulated the production of acetic acid in the gut. Acetic acid is the major SCFA produced by most faecal bacteria and serves as an important pH regulator in the colon, helping to maintain colonic homeostasis. 3. Regulation of gut microbiota composition: The positive modulatory effects of grapefruit CPFEs on intestinal microecology were validated in a simulated human intestinal model. 4. Bioactive constituent basis: HPLC-DAD analysis revealed that grapefruit contained the highest content of naringin (52.03 ± 0.51 mg/g DW), while chachiensis contained the highest content of hesperidin (43.02 ± 0.37 mg/g DW). Both naringin and hesperidin exhibited dose-dependent effects on the growth of Bifidobacterium strains. 5. Prebiotic potential: The findings highlight the biological functions of CPFEs as prebiotic agents, suggesting their potential in food and biomedical applications.	[37]

Beyond these conserved molecular pathways, the antioxidant activity of *C. maxima* exhibits pronounced tissue specificity and developmental dependence. Sarkar et al. [30] indicated that the antioxidant capacities of pulp and peel show non-linear fluctuations, initially decreasing and then increasing, during fruit ripening, with phenolics and flavonoids being the major contributors in the pulp, indicating precise metabolic reprogramming in response to oxidative stress across tissues and developmental stages. Additionally, *C. maxima* peel essential oil has been shown to possess remarkable antioxidant activity against lipid oxidation, with some reports even indicating superior efficacy to the synthetic antioxidant BHT [29]. Unlike the enzyme-system activation mechanism of flavonoids, the terpenes in essential oil mainly act through physical interruption of lipid peroxidation chain reactions, via radical trapping, representing a transcription-independent “immediate defence” mechanism.

### 4.2. Anti-Inflammatory Activity

The anti-inflammatory properties of *C. maxima* have been attributed primarily to its flavonoid constituents, particularly naringin and naringenin, which modulate multiple inflammatory signalling cascades. Mechanistically, these compounds suppress the phosphorylation cascade of the NF-κB pathway, thereby reducing the release of pro-inflammatory cytokines such as TNF-α, IL-1β and IL-6, while concomitantly upregulating anti-inflammatory mediators such as IL-10 (Figure 2B) [37]. In addition, they interfere with the MAPK and COX-2 pathways, contributing to the alleviation of chronic low-grade inflammation associated with metabolic disorders. The synergistic interplay between flavonoids and other phenolic constituents further amplifies the overall anti-inflammatory efficacy, reinforcing the potential of *C. maxima* extracts as functional ingredients for inflammation-related health applications (Figure 2B; Table 2).

### 4.3. Antimicrobial Activity

The broad-spectrum antimicrobial activity of *C. maxima* bioactive components, encompassing bacteria, fungi, and yeasts, has been validated against multiple pathogens (Table 2) [31]. Notably, this broad efficacy does not originate from a single highly potent compound but rather from a “dual-modal” chemical defence system constituted by two functionally distinct groups, lipophilic volatile terpenes (dominated by d-limonene) and hydrophilic flavonoids, operating synergistically through “spatial layering and complementary targeting”. Based on the site of action, this antimicrobial network can be dissected into three logical tiers.

First tier: physicochemical disruption of the cell membrane barrier by lipophilic terpenes. As the principal component of *C. maxima* peel essential oil (accounting for >90%), d-limonene, with its high hydrophobicity (log P ≈ 4.38), preferentially inserts into the hydrophobic core of microbial membrane phospholipid bilayers. This insertion triggers two cascading effects: (1) direct physical disruption, perturbing ordered lipid packing, increasing membrane fluidity, compromising membrane integrity and causing non-selective leakage of K^+^, ATP and small-molecule metabolites, thereby collapsing the transmembrane proton motive force and oxidative phosphorylation coupling; and (2) indirect functional interference, where accumulated terpene molecules alter membrane surface charge and hydrophobic microenvironment, leading to conformational destabilization and catalytic decline of membrane-integrated proteins such as respiratory chain dehydrogenases and ATP synthase [31,38]. This tier relies not on specific receptors but solely on physicochemical compatibility, achieving a broad-spectrum, rapid “physical membrane attack” that underpins the fast bactericidal action of essential oils.

Second tier: precise interference with multiple intracellular biochemical nodes by hydrophilic flavonoids. Unlike the membrane-localized d-limonene, naringin and naringenin, after penetrating the membrane barrier, enter the cytoplasm and exert inhibitory effects through three parallel biochemical pathways: (1) energy metabolism blockade, competitive binding to the active sites of key dehydrogenases in the respiratory chain (e.g., NADH-cytochrome c reductase), impeding electron transport and ATP production; (2) interference with genetic information replication, naringenin binds to DNA gyrase or topoisomerase IV, hindering DNA supercoiling relaxation and replication segregation, thereby suppressing proliferation [32]; and (3) modulation of quorum sensing (QS) and virulence expression, downregulating the synthesis/transduction of QS signal molecules (AHLs or AI-2), inhibiting early biofilm colonization and maturation, and attenuating the coordinated secretion of extracellular proteases, haemolysins and other virulence factors [32,38]. Although this tier is slower in onset, it simultaneously weakens the survival and pathogenic potential of pathogens from three dimensions: energy supply, genetic replication, and collective pathogenicity, embodying the unique “anti-virulence” advantage of flavonoids.

Third tier: spatial synergy and functional amplification. The above two mechanisms are not independent but operate synergistically through a temporal sequence of “membrane disruption first, penetration second” and functional coupling of “efflux inhibition, retention enhancement”. Specifically, d-limonene-induced membrane hyperpermeability creates chemical entry ports for intracellular penetration of flavonoids; meanwhile, flavonoid-mediated suppression of energy metabolism and the QS system markedly impairs microbial membrane repair capacity and the active efflux efficiency of multidrug resistance pumps (such as AcrAB-TolC), thereby reversing or delaying the emergence of drug resistance [38]. Moreover, chemotype differences in the d-limonene/flavonoid ratio among essential oils from different origins directly modulate the relative intensity of each action node, leading to significant fluctuations in overall antimicrobial potential with variety and cultivation environment [38].

### 4.4. Antitumor Activity

The antitumor potential of *C. maxima* has garnered increasing attention, with its active material basis encompassing flavonoids (naringin, naringenin), limonoid triterpenoids (limonin, nomilin) and macromolecular polysaccharides. Unlike single-component synthetic anticancer agents, *C. maxima*-derived natural products exert regulatory effects on multiple key events during tumour initiation, proliferation, invasion and metastasis through an integrated strategy of multi-component synergy and multi-target intervention (Table 2, Figure 2D) [9,33]. Based on the differences in action targets and mechanistic hierarchies, the antitumor effects can be summarized into the following progressive levels.

Direct inhibition of cancer cells by flavonoids and limonoids. Naringenin, as a flavanone aglycone, has been indicated in multiple cancer cell lines to simultaneously inhibit cell growth, proliferation, and migration, and significantly induce apoptosis [9]. Naringin, despite being a glycoside, also exhibits independent antitumor activity, primarily by modulating intracellular reactive oxygen species (ROS) levels and triggering cell-cycle arrest (predominantly at the G2/M phase) [33]. Among limonoids, nomilin shows particularly pronounced inhibitory effects on leukaemia cell proliferation, and the anti-leukaemic effect of crude *C. maxima* fruit extracts correlates significantly with their nomilin content [9]; limonin, together with nomilin, as the major bitter triterpenoid in *Citrus*, has been reported to possess chemopreventive and anticancer activities, involving regulation of phase I/II metabolizing enzymes and interference with the NF-κB-mediated inflammatory pathway [9].

Indirect antitumor potential of crude extracts and seed extracts. Beyond the purified monomers mentioned above, whole-component crude extracts have also indicated anticancer effects in vitro and in animal models. For instance, *C. maxima* peel extract can selectively inhibit breast cancer cell proliferation by upregulating ROS levels [33]; *C. maxima* seed extract exerts significant analgesic and anti-inflammatory effects in animal models, and through effective modulation of the chronic inflammatory microenvironment, it indirectly suggests potential value in suppressing inflammation-associated tumour initiation and progression [16]. Although the evidence at this level is still indirect, it reinforces the multi-layered possibilities of different *C. maxima* tissues in cancer chemoprevention.

The aforementioned components share several intersecting molecular logics in their antitumor effects, including ROS-mediated selective pro-oxidant killing (triggering lethal thresholds in tumour cells), re-locking of cell-cycle checkpoints, and synergistic activation of apoptosis/autophagy pathways. However, clear pharmacokinetic bottlenecks remain. Despite the growing body of evidence from in vitro and animal studies, clinical translation of *C. maxima* bioactive components is still constrained by two major obstacles: (1) low bioavailability, flavonoid glycosides such as naringin have poor membrane permeability and require intestinal microbial metabolism after oral administration, with substantial inter-individual variability; limonoids, on the other hand, are overly lipophilic and insufficiently water-soluble, resulting in peak plasma concentrations that hardly reach in vitro effective doses [9]; and (2) mechanistic network complexity, although multi-target action confers broad-spectrum antitumor coverage, it poses considerable challenges for the identification of preferred targets and the standardized design of dose–response relationships, limiting the development efficiency as single lead agents [9].

### 4.5. Antidiabetic Activity

**Figure 3:**
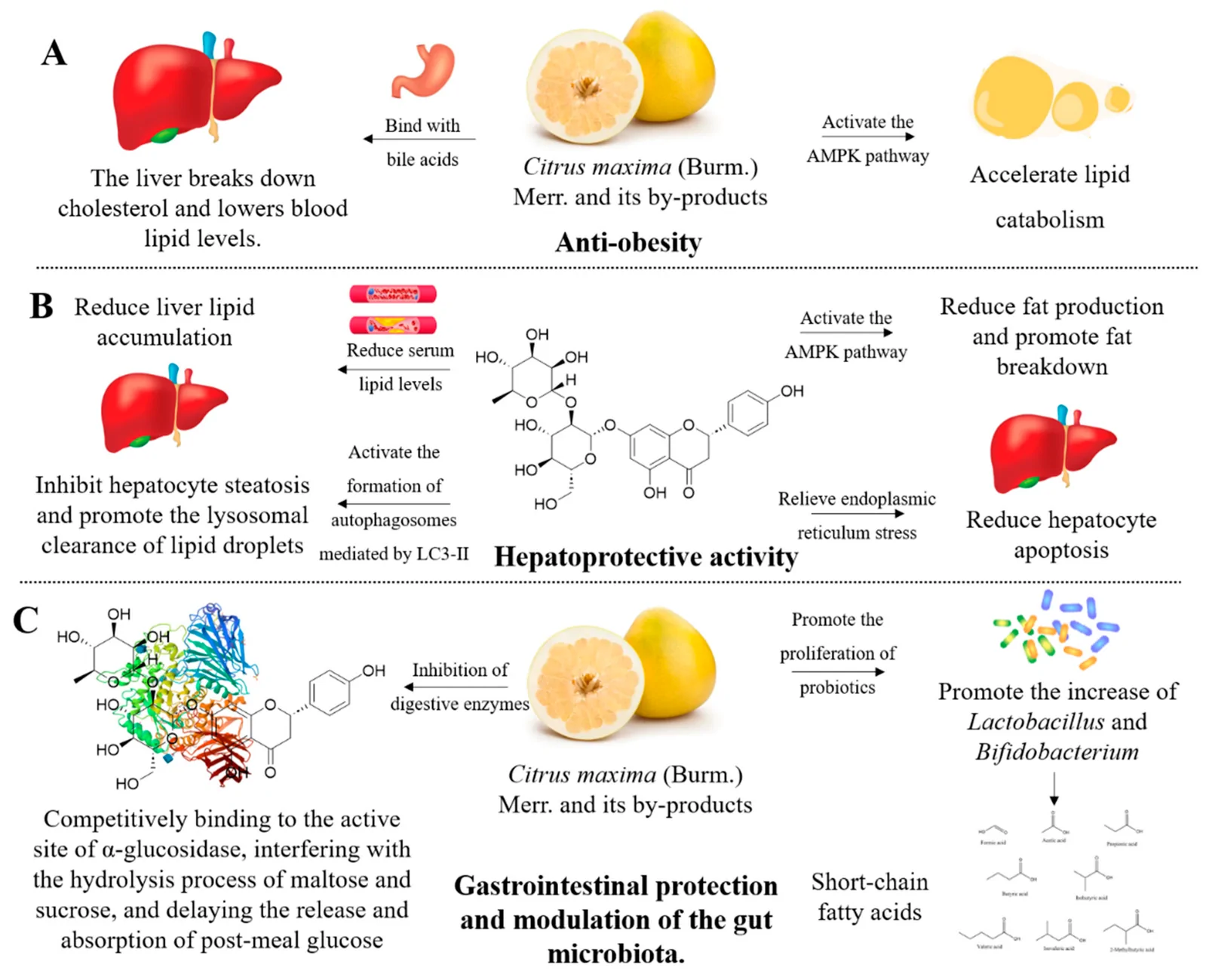
(**A**) Anti-obesity activity, (**B**) hepatoprotective activity, and (**C**) gastrointestinal protection and modulation of the gut microbiota.

The glycaemic regulatory potential of *C. maxima* has drawn considerable research interest (Figure 3A; Table 2). Accumulating evidence indicates that bioactive constituents such as polyphenols and dietary fibre exert hypoglycaemic effects through multiple pathways. In vitro enzyme assays reveal that *C. maxima* juice inhibits α-glucosidase activity with an IC_50_ value of 136.29 µg/mL [6]. Animal studies further suggest that oral administration of *C. maxima* peel extract in streptozotocin-induced diabetic rats significantly lowers fasting blood glucose in a dose-dependent manner [34,39]. These effects involve three levels: (1) inhibition of α-amylase and α-glucosidase activities, delaying postprandial glucose spikes [6]; (2) modulation of insulin secretion and improvement of insulin sensitivity [34]; and (3) protection of pancreatic β-cells against oxidative damage [39].

Naringin, the most abundant flavonoid in *C. maxima*, has been indicated to ameliorate insulin resistance and regulate glucolipid metabolism [32]. In addition, naringin and neohesperidin promote glucose consumption and utilization, and exert preventive and alleviating effects on diabetic-associated cognitive impairment [34]. Notably, the dietary fibre content in *C. maxima* peel is remarkably high, with total dietary fibre in the peel of ‘Jinyou’ cultivars reaching 65.72% [6], which can also improve glycaemic control by delaying gastric emptying, increasing satiety and modulating gut microbiota. The low glycaemic index of *C. maxima* (GI ≈ 25) is closely linked to the fibre-mediated retardation of sugar absorption [34,39].

### 4.6. Anti-Obesity Activity

The potential of *C. maxima* in obesity management has attracted attention. Various parts of the fruit, including pulp, peel, segment membrane and essential oil, have been reported to alleviate obesity and hyperlipidaemia, with mechanisms primarily involving the AMPK–SREBP–PPARs signalling cascade (Table 2; Figure 3A) [5]. Specifically, AMPK, as a cellular energy sensor, is phosphorylated and activated by *C. maxima* flavonoids, which in turn phosphorylate and inhibit the nuclear translocation and activity of its downstream transcription factor SREBP-1c, thereby downregulating the expression of lipogenic genes such as fatty acid synthase (FAS) and acetyl-CoA carboxylase (ACC). Concurrently, AMPK activation enhances the transcriptional activity of PPARα, promoting fatty acid β-oxidation and accelerating lipid catabolism. *C. maxima* peel extract can also independently activate PPARα and GLUT4 pathways, participating in lipid metabolism regulation through promoting glucose uptake and fatty acid oxidation [40]. Bonku et al. [5] identified 14 flavonoids from *C. maxima*, of which naringin, naringenin and other major constituents exert anti-obesity potential via the above pathways.

The role of dietary fibre in anti-obesity is equally noteworthy. *C. maxima* dietary fibre (especially insoluble fibre from the peel) possesses excellent water-holding, oil-holding and bile-acid-binding capacities, which inhibit pancreatic lipase activity and reduce the digestion and absorption of dietary fats [40]. Mechanistically, dietary fibre forms a highly viscous gel in the gastrointestinal tract, delaying gastric emptying and reducing the contact efficiency between nutrients and digestive enzymes; concurrently, by binding bile acids and increasing their faecal excretion, it drives hepatic cholesterol consumption for compensatory bile acid synthesis, thereby indirectly lowering serum lipid levels. High-pressure processing and other technologies can modify the fibre ultrastructure, increasing surface area and exposed hydrophobic groups, further enhancing these functional properties [5]. Moreover, dietary fibre and polyphenols from *C. maxima* by-products modulate gut microbiota composition, for example, increasing the Bacteroidetes/Firmicutes ratio and promoting the proliferation of beneficial bacteria such as *Lactobacillus* and *Bifidobacterium*, thereby facilitating the production of short-chain fatty acids (SCFAs, predominantly acetate, propionate and butyrate), which exert anti-obesity effects at the metabolic regulatory level through activation of G-protein-coupled receptor signalling pathways [32].

### 4.7. Hepatoprotective Activity

The liver is the central organ for lipid metabolism and detoxification, and naringin, as the most abundant flavonoid in *C. maxima*, exhibits pharmacological activity in ameliorating liver injury. In non-alcoholic fatty liver disease (NAFLD) models, the hepatoprotective effects of naringin involve multi-level molecular regulation (Table 2). At the lipid metabolism level, naringin exerts core regulatory effects through activation of AMPK. Activated AMPK phosphorylates and inhibits the nuclear translocation of SREBP-1c, downregulating lipogenic genes (FAS and ACC) and reducing de novo fatty acid synthesis in the liver; at the same time, AMPK activation enhances PPARα-mediated fatty acid β-oxidation, upregulating CPT1a expression and accelerating mitochondrial uptake and oxidative degradation of long-chain fatty acids (Figure 3B). In addition, naringin alleviates hepatocyte apoptosis by relieving endoplasmic reticulum stress (inhibiting the overactivation of the PERK/eIF2α/CHOP pathway) and reduces oxidative mitochondrial damage by lowering ROS levels [5]. Combined supplementation with ‘Shatian’ *C. maxima* flavonoids and dietary fibre further reinforces these effects, reducing serum lipid levels while attenuating hepatic lipid accumulation [10]. Other studies report that flavonoids from ‘Huyou’ peel suppress hepatocyte steatosis and promote lysosomal clearance of lipid droplets by inducing autophagy, specifically, activating LC3-II-mediated autophagosome formation and facilitating p62/SQSTM1 degradation [5]. Ethanolic extracts of *C. maxima* fruit also improve hepatic lipid accumulation in vitro [35].

From a mechanistic network perspective, the hepatoprotective efficacy of *C. maxima* bioactive components does not rely on a single pathway but rather on cross-talk among the AMPK–SREBP–CPT1a lipid metabolic axis, the Nrf2–ARE antioxidant defence system, endoplasmic reticulum stress relief, and the autophagy–lysosomal clearance system, generating synergistic effects across transcriptional regulation, energy metabolism and cellular homeostasis [5,10].

### 4.8. Gastrointestinal Protection and Gut Microbiota Modulation

*C. maxima* by-products (peel, albedo and spongy layer) are rich in dietary fibre, pectin, flavonoids and essential oils, all of which collectively support gastrointestinal health and digestive function through multiple synergistic mechanisms (Figure 3C; Table 2) [9]. Gut microbiota modulation is a core mechanism, involving multi-level crosstalk among “components–microbiota–metabolites–host”. Dietary fibre and flavonoids from *C. maxima* by-products selectively provide carbon sources and growth substrates for beneficial bacteria such as *Lactobacillus* and *Bifidobacterium*, promoting their ecological niche expansion while restraining colonisation by opportunistic pathogens (e.g., Enterobacteriaceae), thereby shaping the overall gut microbial composition [37]. In fermented *C. maxima* products, polyphenols undergo biotransformation, including ring-opening, dehydroxylation and ester hydrolysis, mediated by gut microbiota, generating low-molecular-weight metabolites with enhanced bioavailability, further potentiating the prebiotic effects [41]. Optimized microbial composition significantly increases the abundance of carbohydrate-active enzymes (CAZymes), facilitating fermentation of undigested polysaccharides, with SCFAs, predominantly acetate, propionate and butyrate, as the terminal products. SCFAs, as signalling molecules, on the one hand diffuse passively into colonic epithelial cells for β-oxidation energy supply; on the other hand, they act as agonists of G-protein-coupled receptors (GPR41 and GPR43), activating downstream MAPK and PI3K/Akt pathways involved in systemic energy homeostasis and immune tolerance maintenance [37,41]. In a loperamide-induced constipation model, intervention with *C. maxima* peel powder significantly elevated total SCFA levels in caecal contents, with individual increases in acetate, propionate and butyrate, and notably improved intestinal transit rate [36]. Moreover, SCFAs can inhibit histone deacetylase (HDAC) activity, regulating the expression of intestinal epithelial tight junction proteins (Occludin, ZO-1) and enhancing gut barrier function at the epigenetic level.

The physicochemical effects of dietary fibre constitute another independent yet intersecting pathway. *C. maxima* dietary fibre (especially insoluble fibre from the peel) possesses a highly branched three-dimensional network structure, functioning through the following means: (1) high water-holding and swelling capacities form a gelatinous matrix in the stomach, delaying gastric emptying and diluting the contact efficiency between digestive enzymes and substrates; (2) exposed hydrophobic regions and anionic groups on the polysaccharide chains confer bile-acid-binding capacity, chelating glycocholic and taurocholic acids, reducing their enterohepatic circulation and forcing hepatic cholesterol consumption for compensatory synthesis, thereby indirectly lowering serum lipid levels [36]; and (3) the coarse particles of insoluble fibre physically stimulate the intestinal wall, increasing faecal bulk and shortening intestinal transit time, exerting a mild laxative effect.

Regarding digestive enzyme inhibition, *C. maxima* flavonoids (especially naringin and naringenin) competitively bind to the active site of α-glucosidase, interfering with the hydrolysis of maltose and sucrose and delaying postprandial glucose release and absorption [9]. This mechanism couples with SCFA-mediated enhancement of glucagon-like peptide-1 (GLP-1) secretion, jointly optimizing postprandial glycaemic responses.

On the gastrointestinal protection front, the anti-inflammatory action of naringin is primarily achieved through inhibition of the NF-κB phosphorylation cascade, reducing the release of pro-inflammatory cytokines (TNF-α, IL-1β and IL-6) while upregulating anti-inflammatory cytokines such as IL-10, thereby preserving the integrity of the intestinal mucosal barrier [37]. In addition, *C. maxima* extracts increase acetylcholinesterase activity in the intestinal myenteric plexus, promoting gastrointestinal motility and improving digestive function [9]. Collectively, these findings underscore the application potential of *C. maxima* and its by-products in functional food development and intestinal health maintenance, with value derived from the synergistic complementarity among the physical modulation of dietary fibre, enzyme inhibition and anti-inflammatory actions of flavonoids, and microbiota-mediated metabolic transformation [9,41].

## 5. Safety and Toxicological Evaluation of *C. maxima* and Its By-Products

The application of *C. maxima* and its processing by-products in functional foods and botanical drug development is expanding rapidly, and its safety evaluation has progressively evolved from empirical understanding to a toxicological research framework [5,42]. Available evidence indicates that *C. maxima* pulp is safe for daily dietary consumption. Sarkar et al. [30], using standard acute oral toxicity and genotoxicity assays (Ames test, micronucleus test, and chromosomal aberration test), suggested that *C. maxima* seed extract exhibited no mortality or overt toxic reactions in animals at doses up to 2000 mg/kg, indicating practically non-toxic characteristics. A review by Sapkota et al. [5] noted that the major flavonoid naringin has an oral LD_50_ value exceeding 5000 mg/kg in rat models, indicating a wide safety margin. In a clinical trial involving obese patients, Nunes et al. [43] observed no significant adverse effects following daily intake of a defined dose of *C. maxima* extract, further supporting its short-term tolerability in humans.

However, the safety risks of *C. maxima* are highly dependent on its chemical composition and exhibit marked cultivar heterogeneity. The core risk stems from the irreversible inhibition of CYP3A4 by furanocoumarins. These compounds undergo metabolic activation to form reactive intermediates that covalently bind to the heme prosthetic group of CYP3A4, leading to irreversible loss of enzymatic activity, with inhibitory effects lasting over 72 h. This can precipitate severe drug–herb interactions with dozens of clinically used drugs, including lipid-lowering agents (atorvastatin, simvastatin), antihypertensives (nifedipine, amlodipine), immunosuppressants (cyclosporine A, tacrolimus), and antiarrhythmics (amiodarone) [5,42]. Substantial inter-cultivar differences exist in furanocoumarin content: the levels in ‘Guanxi Honey’ and ‘Liangping’ *C. maximas* far exceed the safety threshold (0.68 mg/L), whereas ‘Shatian’ and ‘Huyou’ are relatively safe [5]. Furthermore, the potential hepatotoxicity of coumarins at high doses (possibly related to CYP2E1-mediated generation of reactive metabolites), the phototoxicity and contact sensitization of terpenes in essential oils, the allergenicity of *C. maxima* proteins (such as Cit s 1 and Cit s 3 allergens) capable of eliciting reactions ranging from mild oral allergy syndrome to severe anaphylaxis, and the dietary exposure risks from pesticide residues and heavy metals (lead, cadmium, arsenic) collectively constitute the multidimensional framework of *C. maxima* safety assessment [42].

Although current research has preliminarily delineated the material basis and mechanisms of the major toxicological risks of *C. maxima*, significant gaps remain in several areas: the establishment of safety-risk fingerprint profiles for different cultivars (encompassing comprehensive spectra of furanocoumarins, coumarins, allergens, and pesticide residues), validation of human pharmacokinetic–pharmacodynamic (PK–PD) correlations, definition of toxicity thresholds for chronic low-dose exposure, and the construction of an integrated risk assessment system based on physiologically based pharmacokinetic (PBPK) modeling [5,42]. Future research should integrate metabolomics, toxicogenomics, and clinical pharmacology approaches to advance *C. maxima* safety evaluation from a static, single-compound, single-target perspective to a systematic, precise assessment paradigm that encompasses multi-component interactions, multi-cultivar coverage, and multi-dimensional integration, thereby providing a robust scientific foundation for its deeper exploitation in the food and pharmaceutical sectors [30,43,44].

## 6. Industrial Applications of *C. maxima* and Its By-Products

In recent years, driven by the national “carbon peaking and carbon neutrality” strategy and the growing adoption of circular economy principles, the high-value utilization of *C. maxima* by-products has transitioned from fundamental laboratory research to industrial practice, giving rise to a comprehensive multi-dimensional utilization framework encompassing health foods, edible packaging materials, pharmaceutical and nutraceutical products, and agricultural inputs.

### 6.1. Health Foods

**Figure 4:**
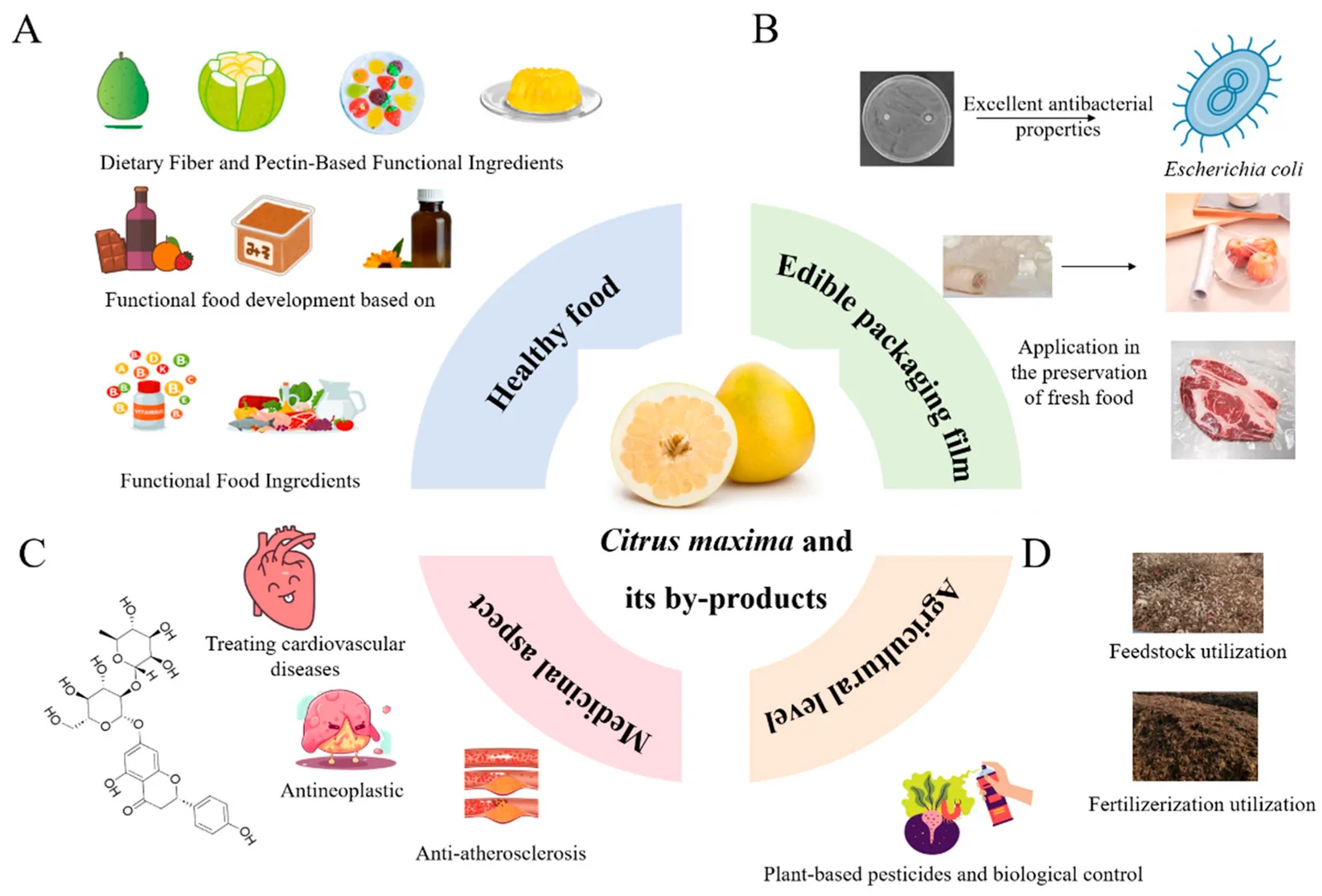
Industrial applications of *C. maxima* and its by-products. (**A**) Health foods, (**B**) edible packaging films, (**C**) pharmaceutical applications, and (**D**) agricultural applications.

Health foods represent the largest and most commercially mature sector in the industrial application of *C. maxima* by-products (Figure 4A). *C. maxima* processing residues are rich in dietary fibre, pectin, flavonoids and a variety of micronutrients, all of which offer broad prospects for functional food development [2,13]. At present, the industrial application of *C. maxima* by-products in the health food domain follows three main technological pathways.

First, dietary fibre and pectin-based functional ingredients. The total dietary fibre content in *C. maxima* peel can exceed 65%, of which soluble and insoluble fractions account for approximately 15% and 43%, respectively [2]. These fibres exhibit excellent water-holding, oil-holding, and swelling capacities, enabling their use as natural functional additives in baked goods, meat products, beverages, and snack foods, thereby enhancing dietary fibre content while improving textural properties [2]. Advanced processing techniques such as ultrafine grinding can further enhance the functional characteristics of *C. maxima* peel dietary fibre. Studies have indicated that ultrafine-ground peel fibre reduces the Firmicutes/Bacteroidetes ratio by 47% in high-fat diet-fed mice while promoting the proliferation of beneficial genera such as *Bifidobacterium* and *Alistipes* [2]. Zhang et al. [45] further reported that *C. maxima* peel dietary fibres of different particle sizes can synergistically stabilize Pickering emulsions through interfacial adsorption, offering a novel technological approach for low-fat emulsified food development. *C. maxima* pectin, as an excellent gelling agent and stabilizer, has been widely applied in food systems such as jams, jellies, and dairy products [2]. Ahmad et al. [46], through formulation-driven rational design, developed a directly compressible chewable tablet by combining *C. maxima* peel fibre with pectin, achieving satisfactory disintegration properties and palatability while maintaining fibre content, thus providing a new carrier for oral delivery of peel dietary fibre.

Second, functional food development based on flavonoid bioactive components. Naringin, the most representative flavonoid in *C. maxima*, possesses multiple physiological benefits including antioxidant, lipid-lowering, and metabolism-promoting effects. Once considered waste, it is now collected by deep-processing enterprises for the extraction of high-purity naringin, with products widely used in food, pharmaceutical, and cosmetic manufacturing. Some companies have expanded their deep-processing product portfolios, with new high-end functional products such as probiotic *C. maxima* juice and medicinal flavonoid extracts currently in the pilot stage.

Third, whole-fruit utilization and novel functional food ingredients. Driven by the “whole-fruit utilization” concept, the health food applications of *C. maxima* by-products are expanding into broader dimensions. The peel, pomace, and seeds, rich in dietary fibre, pectin, and polyphenols, provide an excellent raw material base for the development of high-value-added functional ingredients [2]. In recent years, the application of *C. maxima* by-products in craft brewing has drawn particular attention, emerging as a new growth point in the *C. maxima* deep-processing industry. *C. maxima* peel (especially the oil gland layer) is rich in volatile terpenes such as limonene and flavonoids such as naringin, which can be added during wort saccharification or fermentation to impart a distinctive citrus aroma and a nuanced bittersweet aftertaste to beer; *C. maxima* juice, rich in fructose, organic acids, and vitamins, can be co-fermented with wort to enrich the flavour profile. At the technological level, semi-solid fermentation enables the graded utilization of the flavedo and segment membrane layers: the peel, after dicing and hardening, serves as a solid-state fermentation packing material, while the juice participates in liquid-state fermentation; intermittent percolation facilitates the deep integration of flavour and functional components, enhancing sensory quality while effectively controlling the release of bitter compounds. In addition, a process route that incorporates dried peel during saccharification with high-temperature cooking to promote full aroma release, followed by low-temperature fermentation with pure *C. maxima* juice, has also been validated to produce craft fruit beers with rich fruity notes and layered complexity. Beyond peel and juice, *C. maxima* flowers, another category of by-product, have been incorporated into brewing; by introducing flower aroma indirectly at appropriate stages, specialty beers with a delicate floral fragrance can be produced. At present, products such as ‘Jinyou’ beer, ‘Liangping’ craft beer and ‘Changshou’ fruit beer have achieved industrial production, with some enterprises having built 10,000-ton-scale craft beer production lines covering both fresh and pasteurized beer product lines. *C. maxima* seeds, as another important by-product, also indicate potential as functional food ingredients; debittered seed powder, rich in biopolymers and proteins, has been successfully applied in the development of low-fat ice cream, serving dual functions as a fat replacer and stabilizer [17]. Furthermore, the prebiotic potential of *C. maxima* by-products in modulating gut microbiota has attracted considerable interest: the pectin and oligosaccharides they contain can be fermented by gut microbes to produce short-chain fatty acids, thereby improving host metabolic health, providing a scientific basis for next-generation functional food development [2]. Currently, several enterprises have developed diversified product matrices around whole *C. maxima* fruit, encompassing functional beverages, craft beers, dietary supplements, and bakery ingredients. With ongoing advances in extraction and modification technologies, the high-value utilization of *C. maxima* by-products is expected to become a significant growth driver in the functional food sector.

### 6.2. Edible Packaging Films

Edible packaging films represent a highly promising emerging direction in the high-value utilization of *C. maxima* by-products. As the environmental pollution caused by conventional petroleum-based plastic packaging becomes increasingly severe, the development of biodegradable, edible green packaging materials has become a research hotspot at the intersection of food science and materials engineering (Figure 4B) [47]. *C. maxima* peel is rich in pectin, cellulose, and flavonoids, making it an ideal raw material for preparing edible/biodegradable packaging films [2].

At the fundamental research level, functional edible packaging materials have been successfully fabricated using *C. maxima* peel powder and carboxymethyl cellulose as the base matrix, with nisin incorporated as an active antimicrobial compound. Under optimized formulation, the resulting films exhibited thickness below 0.2 mm, oxygen permeability below 1.3 meq·kg^−1^, water vapour transmission rates in the range of 21.7–22.9 g·m^−2^·h^−1^, and mechanical strength of 3.4 MPa; additionally, the complex composition of the peel powder imparted antioxidant activity of approximately 130 µmol Trolox·g^−1^ [48,49]. These composite packaging materials indicated successful antimicrobial activity against both *Bacillus subtilis* (Gram-positive) and *Escherichia coli* (Gram-negative) [50]. Other studies have optimized the formulation of edible films based on *C. maxima* peel slurry, with optimal conditions identified as 5% peel slurry, 1% glycerol, and 0.4% sodium alginate [51].

At the application level, *C. maxima* peel-based edible films have shown practical value in fresh food preservation. *C. maxima* pectin edible films significantly reduced juice loss from lean pork, retarded pH elevation, slowed protein degradation, and inhibited microbial growth, extending refrigerated shelf life by 2–4 days, with preservation effects slightly superior to those of polyethylene cling film [2]. Environmentally friendly composite films based on chitosan as the carrier and ‘Changshan Huyou’ peel extract as the active ingredient significantly extended the shelf life of grapes at room temperature while effectively reducing fruit decay rates [47]. Dual-ultrasound-assisted technology enables the simultaneous recovery of pectin and essential oil from *C. maxima* peel for the programmable assembly of antimicrobial biodegradable films [49]. Collectively, these studies indicate that *C. maxima* peel-based edible packaging films not only achieve the resourceful utilization of agricultural by-products but also offer green and sustainable solutions for the active food packaging sector [50].

### 6.3. Pharmaceutical Applications

The pharmaceutical development of *C. maxima* bioactive components represents the highest-value-added direction in its industrial applications [2]. Among them, naringin, the most abundant flavonoid in *C. maxima*, has indicated a clear translational pathway from basic pharmacological research to novel drug development (Figure 4C) [5,15]. The pharmacological activities of naringin have been extensively validated, with its intervention spectrum covering major chronic diseases including cardiovascular disorders, cancer, metabolic diseases, skeletal disorders, and neurodegenerative conditions [5,30]. In the cardiovascular system, naringin exerts multiple effects, including lipid regulation, anti-atherosclerosis, blood pressure reduction, inhibition of cardiac hypertrophy, anti-myocardial infarction, attenuation of ischaemia/reperfusion injury, and amelioration of pulmonary hypertension, through signalling axes such as PI3K-Akt-mTOR, p-eNOS/p-Akt/p-ERK, and miR-126/GSK-3β/β-catenin [10,15]. In oncology, naringin complexes effectively inhibit aberrant activation of the PI3K-Akt-mTOR pathway, thereby inducing apoptosis in hepatocellular carcinoma cells, suggesting its potential as a candidate for tumour intervention [10]. Moreover, naringin shows outstanding performance beyond the cardiovascular arena: in vivo serum metabolomics studies have revealed that it significantly ameliorates osteoporosis symptoms and endogenous metabolic disturbances in ovariectomized mice; concurrently, mechanism-based investigations focusing on the DRP1/LRRK2/MCU axis have further uncovered its distinctive neuroprotective effects in cerebral ischaemia/reperfusion injury models. In summary, naringin exhibits broad-spectrum and specific bioactivities across multiple pathological models through synergistic multi-target, multi-pathway regulation, and this multi-level evidence provides a solid experimental foundation for its further development and clinical translation [5,52].

### 6.4. Agricultural Applications

The agricultural application of *C. maxima* by-products constitutes a critical link in achieving intra-industry circularity and zero-waste throughout the entire value chain, primarily encompassing three directions: feed utilization, fertilizer utilization, and botanical pesticide development (Figure 4D).

Feed utilization. *C. maxima* peel pomace is rich in pectin, cellulose, and a variety of bioactive polyphenols. Its development as a non-conventional feed resource offers dual benefits: alleviating the competition between humans and livestock for cereal grains and reducing the waste disposal pressure from the citrus processing industry. However, its high moisture content, high fibre level, and the presence of bitter compounds limit direct feeding efficiency, necessitating appropriate pretreatment to improve nutritional accessibility and palatability [53,54]. In terms of precision nutritional regulation, it has been documented that incorporating 2% *C. maxima* peel powder into broiler diets not only retards lipid oxidation of intestinal contents through its abundant phenolic compounds but also significantly suppresses the proliferation of pathogenic *Escherichia coli* and *Staphylococcus*, thereby improving gut microecological balance [4]. Regarding technological processing routes, ensiling fermentation is considered the most economical and effective approach for large-scale feed conversion of *C. maxima* peel pomace. Studies have shown that precise control of material moisture content to 70% (wet basis), supplementation with 2% urea to compensate for nitrogen deficiency, inoculation with Effective Microorganisms (EM) at 2.5 mL·kg^−1^, and co-addition of bamboo powder as an adsorbent and structural regulator can effectively inhibit butyric acid fermentation, yielding silage with an optimal lactic acid/acetic acid ratio [4,53]. Furthermore, mild fermentation of fresh discarded *C. maxima* has been indicated to partially degrade anti-nutritional factors such as naringin and increase crude protein content, showing favourable feed intake and digestive metabolic performance in both ruminant and monogastric feeding trials [55].

Fertilizer utilization. *C. maxima* processing by-products (e.g., peel and pomace) are rich in organic matter, mineral nutrients, and functional bioactive substances, making them highly promising raw materials for organic fertilizers. Studies indicate that *C. maxima* peel can serve as a functional component of disease-preventive specialty fertilizers for citrus [55,56]. Bio-organic fertilizers prepared through synergistic fermentation of peel with straw, livestock manure, and other auxiliary materials, using EM agents and cellulolytic microbes, not only provide balanced macronutrient and micronutrient supply for crop growth but also enhance soil ecological functions by modulating soil bacterial community structure and increasing the abundance of beneficial taxa [57,58]. Field trials have further suggested that by-product-based organic substitution strategies synergistically optimize fruit sugar-acid ratios and phenolic metabolism, significantly improving the flavour quality and nutritional value of *C. maxima* [24,59]; rational combined application of organic fertilizers can also maintain or improve overall fruit quality under reduced chemical fertilizer conditions, thereby lowering the environmental risks of chemical inputs [22]. This fertilizer pathway not only effectively absorbs orchard waste fruits and processing residues, alleviating environmental pollution pressure, but also establishes a closed-loop recycling system of “waste → organic fertilizer → tree health”, aligning with the core tenets of green and sustainable agricultural development.

Botanical pesticides and biological control. *C. maxima* processing by-products (especially the peel) are rich in flavonoids, essential oils, and limonoids, which exhibit broad-spectrum antimicrobial and insecticidal bioactivities, constituting a valuable natural resource reservoir for the development of environmentally friendly botanical pesticides [58,60,61]. In terms of antimicrobial activity, ethanolic extracts of *C. maxima* peel and their ethyl acetate fractions show significant inhibitory effects against various plant pathogenic fungi. Naringenin, a representative endogenous flavanone, not only activates systemic acquired resistance by modulating endogenous hormone homeostasis and transcriptional reprogramming of defence-related genes in tobacco to enhance protection against *Phytophthora nicotianae* (*P. nicotianae*), but also directly inhibits mycelial growth and sporangium production of *P. nicotianae*, while exhibiting excellent control efficacy against root rot diseases caused by *Fusarium* spp. and *Pythium* spp. Beyond antifungal activity, *C. maxima* peel essential oil, rich in terpenes such as limonene and α-terpineol, has been suggested to possess significant contact and fumigant toxicity against various stored-grain pests and sanitary insects [12]; its extracts also display potent larvicidal activity against Aedes aegypti and Aedes albopictus [23,61]. Additionally, biochar prepared from *C. maxima* peel shows promising application prospects in environmental remediation fields such as soil disinfection and water purification.

## 7. Conclusions and Future Perspectives

This Review provides a comprehensive overview of current research on *Citrus maxima* (*C. maxima*), covering the nutritional and chemical profiles of its fruit and processing by-products, the structural diversity and chemotaxonomic significance of its secondary metabolites, the molecular mechanisms underlying its major bioactivities, and its safety evaluation and industrial application landscape. We further critically examine the practical applications and developmental prospects of *C. maxima* and its derivatives in food processing, pharmaceuticals and nutraceuticals, edible packaging, and agricultural inputs.

Despite substantial data on chemical characterization, functional evaluation, and preliminary product development, several pivotal scientific questions and technological bottlenecks must be addressed to enable efficient resource utilization and sustainable whole-value-chain development. These are grouped below by thematic logic. (i) Biosynthesis and metabolic engineering (metabolic/cardiovascular and chemotaxonomic relevance). Multi-omics integration, genomics, transcriptomics, and metabolomics, should decipher the biosynthetic pathways of characteristic secondary metabolites such as naringin, limonoids, and furanocoumarins, and identify key enzyme-encoding genes and upstream regulatory elements. This will supply genetic resources and theoretical support for marker-assisted breeding of elite cultivars and green bio-manufacturing of bioactive components. (ii) Green extraction and purification. Given the chemical diversity of *C. maxima* constituents, efforts should target efficient, environmentally friendly, and cost-effective extraction and purification systems, overcoming industrial-scale bottlenecks for high-purity naringin, pectin, and essential oil fractions, thereby enhancing fractional utilization efficiency and economic added value across food, pharmaceutical, and agricultural sectors. (iii) Mechanistic dissection and target validation (cytoprotective/oncological and cardiometabolic axes). Current understanding of bioactivity largely rests on crude extracts or preliminary single-pathway validation. Future work should deploy gene knockout/knock-in animal models, CRISPR-based screening, and single-cell sequencing to delineate molecular targets and regulatory networks of key active components. Well-designed randomized controlled trials are urgently needed to verify health benefits and safety, shifting the paradigm from descriptive phenomenology to causal-chain verification linking “target → pathway → phenotype”. (iv) Germplasm evaluation and standardized cultivation. Geographical variation in cultivation conditions markedly affects quality attributes. Future work should investigate quality formation patterns of major cultivars across ecological zones and establish standardized cultivation systems, nursery propagation, planting, water and nutrient management, and pest and disease control, ensuring a stable supply of high-quality raw materials. (v) Post-harvest preservation and storage/transport. Fruits are susceptible to moisture loss, pulp dehydration, decay, and flavour deterioration, limiting shelf life and economic value. Research should prioritize green preservation technologies, including plant-derived antimicrobial coatings, intelligent packaging, controlled-atmosphere storage, and precision cold-chain management, to reduce losses and enhance market competitiveness. (vi) Diversified high-value product development. Industrial application of by-products remains largely confined to low-value products such as dietary fibre and pectin, whereas high-value products—naringin, essential oils, and pectin oligosaccharides—remain in their infancy. Future efforts should pursue a “whole-fruit zero-waste” goal, expanding into craft brewing, functional fermented beverages, edible packaging films, botanical pesticides, and biomedicine to construct a high-value product portfolio. (vii) Full-chain industrialization and standardization. The processing industry faces non-uniform processes, low efficiency, and weak quality control. A comprehensive standardized management system is imperative, covering raw material quality grading, extraction process specifications, product quality standards, and safety evaluation criteria, providing institutional support for standardized operations and quality assurance.

In summary, *C. maxima* represents a citrus fruit with multifaceted nutritional, functional, and economic value. Research along the entire industrial chain, from germplasm development to high-value by-product utilization, has begun to take shape, yet substantial space and innovative potential remain, spanning systems-biology-level mechanistic elucidation to industrial-scale technological integration. Future efforts should integrate phytochemistry, molecular biology, food engineering, clinical nutrition, and materials science to drive *C. maxima* research from linear, single-component, single-target understanding toward a paradigm embracing multi-component interactions, multi-target synergy, and multi-dimensional applications, providing a robust scientific foundation for green, sustainable industry development and the transition toward a circular economy.

## 8. Review Methodology

This systematic review followed the Preferred Reporting Items for Systematic Reviews and Meta-Analyses (PRISMA) guidelines. A comprehensive literature search was performed in Scopus, Web of Science, PubMed, and Google Scholar to identify studies reporting on the natural compounds, nutritional composition, bioactive properties, and applications of *Citrus maxima* and its by-products. The literature analysis covered the period from January 2000 to May 2026, with earlier foundational publications included when they were essential for taxonomic, botanical, or traditional medicinal contexts. The search strategy combined terms related to the plant and its by-products with terms related to chemical composition, biological activity, and application, including “*Citrus maxima*”, “pomelo”, “*Citrus* grandis”, “peel”, “flavedo”, “albedo”, “segment membrane”, “seed”, “by-product”, “flavonoid”, “limonoid”, “coumarin”, “alkaloid”, “essential oil”, “naringin”, “antioxidant”, “anti-inflammatory”, “antitumor”, “antidiabetic”, “functional food”, and “industrial application”. Boolean operators (AND, OR, NOT) were used to refine the search. Only peer-reviewed articles published in English were considered. After removal of duplicates, titles and abstracts were screened independently by two reviewers, and full texts of potentially eligible studies were assessed against predefined inclusion and exclusion criteria. Data were extracted into a standardized form, including plant cultivar, tissue or by-product, extraction method, chemical compounds identified, biological activities, experimental models, and key findings. Disagreements were resolved through discussion or consultation with a third reviewer. The methodological quality of included studies was assessed using appropriate tools, and the results were synthesized narratively and, where possible, tabulated to highlight chemical diversity, tissue-specific distribution, pharmacological activities, and industrial translation potential. This systematic approach ensures transparency, reproducibility, and reliability of the evidence presented in this Review.

## Data Availability

No new data were created or analyzed in this study. Data sharing is not applicable to this article.
